# One-hour Recovery Time in Subjects Undergoing Percutaneous Liver Biopsy: A Quality Improvement and Patient Safety Project

**DOI:** 10.7759/cureus.5799

**Published:** 2019-09-29

**Authors:** Billy-Joe Liane, Guy Dooley, Sonali Sarkar, Angelo Paredes, Amilcar L Morales-Cardona

**Affiliations:** 1 Internal Medicine, Brooke Army Medical Center, San Antonio, USA; 2 Gastroenterology and Hepatology, Brooke Army Medical Center, San Antonio, USA

**Keywords:** liver biopsy, complications of liver biopsy, ultrasound guided liver biopsy, non-alcoholic fatty liver disease (nafld)

## Abstract

Introduction

Liver biopsy is the gold standard in diagnosing, staging and guiding clinical management in liver disease. There are currently no standard guidelines for liver biopsy recovery time. The aims of this project are to study the safety of a one-hour recovery time after percutaneous liver biopsies and to measure the rate of complications and identify risk factors.

Methods

A total of 500 consecutive subjects who underwent a percutaneous liver biopsy at a single-center teaching institution (Brooke Army Medical Center) were enrolled between December 2016 and October 2018. Biopsies were performed using a 14-gauge Bard® Monopty® core biopsy needle using bedside ultrasound. Complications were defined as: Pain level > 5 out of 10, hospitalizations, emergency department visits, or other. Major complications were defined as: hospitalizations and emergency department visits.

Results

The only complication that required hospitalization was identified during the first hour of recovery. Liver biopsies of subjects with body mass index (BMI) ≥35 kg/m^2^ were not associated with more complications when compared to patients less than 30 kg/m^2^. Using a spinal needle (3.5’’) to anesthetize the liver capsule in subjects with excess subcutaneous tissue did not result in more complications when compared to the standard 1.5’’ needle. Only 3% of the patients who received lidocaine alone for the biopsy required post-procedure medications.

Conclusion

Ultrasound-guided percutaneous liver biopsies, using a 14-gauge needle, were overall found to be safe. A one-hour post recovery period is adequate to identify all immediate major complications.

## Introduction

As the prevalence of chronic liver disorders such as non-alcoholic fatty liver disease (NAFLD) continues to increase, there has been some interest in evaluating different approaches to decrease the financial burden and improve access to care for these patients. To date, liver biopsy is the gold standard for the diagnosis and staging of multiple liver disorders. Overall, liver biopsy is a safe procedure, but still carries the risk of several complications to include bleeding, infection, severe pain, bowel perforation, and gallbladder injury. The overall risk of serious complications is around 1%, with death directly related to liver biopsy estimated around 1 in 10,000 biopsies [1-3]. The complication rate varies based on the indication, with a higher rate of complications in patients under evaluation of liver masses and those with abnormal coagulation parameters [4].

For most practitioners, liver biopsies are time-consuming and occasionally a burden for the gastroenterology suites as patients require constant monitoring and prolonged recovery times. Currently, there is no national standard on post-biopsy recovery times. Most published studies report recovery times ranging from two to six hours with the majority of hepatologist following the American Association for the Study of Liver Diseases (AASLD) Liver Biopsy practice guideline recommendation of two to four hours [5]. There is limited data regarding shorted recovery times. Nodarse-Perez et al. demonstrated in a randomized controlled clinical trial that there is no difference in complication rates between two hours and six hours post-liver biopsy [6]. In 2007, Beddy et al. demonstrated that a one-hour post-liver biopsy recovery time was feasible and safe for most patients undergoing liver biopsy by interventional radiology [7]. Only one study, published in 2005, addressed the possibility of decreasing recovery time to one hour. The study by Firpi et al. was able to detect most major complications during the first hour post-liver biopsy with a safety profile that was similar to extended recovery times [8]. As we continue to see an increase in the prevalence of chronic liver diseases, there is a need to explore possible interventions to address cost, patient access, and adequate use of space for this intervention without compromising patient care and safety.

Our study specifically examines recovery time after percutaneous ultrasound-guided liver biopsies. The purpose of study is to assess the safety of a one-hour post-liver biopsy recovery time, which could potentially save time for the patients and ancillary staff as well as improve turn-over and increase patient access.

## Materials and methods

The study aimed to prospectively evaluate the safety of a one-hour liver biopsy recovery time in subjects undergoing a clinically indicated liver biopsy at a single teaching institution (Brooke Army Medical Center, San Antonio TX).

Adult men and women over the age of 18 who required a percutaneous liver biopsy were enrolled between December 2016 and October 2018. Both inpatients and outpatients were included in this study. Patients with contraindications for percutaneous liver biopsy, i.e., vascular liver tumors, severe coagulopathy, or severe thrombocytopenia (platelets less than 50,000 per microliter), were excluded from the study. All percutaneous biopsies were performed using a 14-gauge Bard® Monopty® core biopsy needle (Bard, Tempe, AZ) after a site was localized using bedside ultrasound. 1% Lidocaine was used for local anesthesia and injected in a dose according to the physician’s discretion, usually ranging from 5-10 milliliters, unless there was a patient drug allergy. Each patient was offered the option of conscious sedation during liver biopsy consisting of fentanyl and midazolam, given at the physician’s discretion. A select number of patients received their liver biopsy after undergoing an already planned and scheduled esophagogastroduodenoscopy or colonoscopy. Patients who underwent an endoscopic procedure had a post-endoscopic procedure recovery time of one hour before their liver biopsy. Biopsies were performed by gastroenterology fellowship trainees and staff physicians. Demographics, procedural technique, and recovery time were recorded by the physician performing the liver biopsy. The post-procedure standard practice involved having the patients rest in the right lateral decubitus position and checking vital signs every 15 minutes by the nursing personnel. Pre- and post-procedure pain assessment was performed by nursing staff and documented in the patient’s chart. Subjects received a phone call by our nursing team 24 hours post-procedure for reassessment. The electronic medical record was reviewed at least one week after the procedure to identify any subjects that presented to the emergency department with procedure-related complications. Complications were defined as pain level > 5 out of 10, hospitalizations, emergency department visits, or other (e.g., vomiting). Major complications were defined as: hospitalizations and emergency department visits directly related to liver biopsy.

Percentages were used for the qualitative variables and quantitative variables were expressed using the standard deviation of means. The Chi-squared test and Fisher’s exact test were performed for comparison of categories. A P value of <0.05 was set for the comparison to be considered statistically significant. The Fisher exact test reported significance for cell counts <5 for comparison of 2 x 2 categories.

## Results

As outlined in Table 1, a total of 500 patients (290 males and 210 females) with a mean age of 54 years were included in our study. The average BMI was 33 and the most common indication for liver biopsy was NAFLD. A prior endoscopy was performed on 125 of the patients before liver biopsy. A spinal needle for application of Lidocaine was used in 104 patients and double pass biopsies were performed in 32 patients. A total of 439 patients used conscious sedation, whereas 61 patients used local anesthesia only (Lidocaine 1%). Gastroenterology fellows, with direct supervision of a gastroenterology staff, performed 366 liver biopsies, with 233 of those liver biopsies performed by first-year gastroenterology fellows. 56.9% of patients had a recovery time of less than one hour. No patients during this study period had greater than two hours of recovery time. The rest of the patients were discharged within 90 minutes post-procedure. The most common indication for the additional time was pain management.

**Table 1:** Demographics and baseline characteristics of subjects SD: Standard deviation; BMI: Body mass index; n: sample size; NAFLD: Non-alcoholic fatty liver disease; PGY: Post-graduate year.

Age, mean (SD)	54 ± (10)
Males (%)	58%
BMI (kg/m²), mean (SD)	33 ± (6)
Indication, n (%)	
NAFLD	420 (84%)
Auto-immune hepatitis	23 (4.6%)
Other	57 (11.4%)
Endoscopy Pre-liver biopsy, n (%)	125 (25%)
Spinal needle use, n (%)	104 (20.8%)
Double pass biopsies, n (%)	32 (6.4%)
Lidocaine only, n (%)	61 (12.2%)
Level of experience, n (%)	
PGY4	233 (46.6%)
PGY5	100 (20%)
PGY6	33 (6.6%)
Staff	134 (26.8%)
Recovery time, n (%)	
≤1 hour	284 (56.9%)
>1, ≤2 hours	215 (43.1%)
> 2 hours	0

With regards to the total complication rate of 6.4% seen in Table 2, the majority of those patients had pain >5/10 on the pain scale (81.25% of all complications and 5.2% of all patients). Less frequent complications included one hospitalization (3.1%), three emergency department visits (9.4%), and two episodes of vomiting (6.25%). Oral-only pain medications were administered in 47 patients (9.4%). Intravenous-only medications were prescribed in 13 patients (2.6%), and three patients received intravenous and oral medications (0.6%). Two patients in the Lidocaine-only cohort received post-procedural pain medications (3.3%).

**Table 2:** All post-procedure complications and additional pain medications administered n: Sample size; ED: Emergency department; Rx: Prescription pain medications; PO: Oral; IV: Intravenous.

All complications, n (%)	32 (6.4%)
Complication type, n (%)	
Pain > 5 out of 10	26 (81.25%)
Hospitalizations	1 (3.1%)
ED visits	3 (9.4%)
Other (vomiting)	2 (6.25%)
Pain (post-procedure), n (%)	
Pain ≤ 5	474 (94.8%)
Pain > 5	26 (5.2%)
Post-procedure Rx administered, n (%)	
PO only	47 (9.4%)
IV only	13 (2.6%)
PO + IV	3 (0.6%)
Post-procedure Rx in Lidocaine-only cohort, number and (%)	2/61 (3.3%)

A comparison was made of the complications rates involving several variables to include BMI, trainee involvement, and endoscopic procedures pre-liver biopsy. This is shown in Table 3. In patients with BMI >35 kg/m² the complications rate was 4.9%, compared to 7.1% with a BMI between 30-34 kg/m² and 7.7% with a BMI <29 kg/m². Hospitalizations and emergency department visits occurred in 0% of patients with a BMI of <29 kg/m², in 1.3% of patients with a BMI of 30-34 kg/m², and in 1% of patients with a BMI of >35 kg/m². Post-procedure prescriptions were given in 10.3% of patients with a BMI of >35 kg/m², 12.9% of patients with a BMI of 30-34 kg/m², and 15.5% of patients with a BMI of <29 kg/m². We evaluated complication rates of fellows performing the liver biopsy versus attending physicians only. The overall complication rate for attending physicians was 11.2% compared to 4.6% in the fellow’s group. Hospitalizations and emergency department visits occurred at a rate of 0.8% with fellows compared to 0.7% with the attending physicians. Post-procedural pain medication prescriptions were given in 14.9% of the cases performed by attending physicians versus 11.7% of the patients undergoing liver biopsy by a fellow. Lastly, endoscopy pre-liver biopsy had a complication rate of 4.8% versus 6.9% in patients undergoing liver biopsy only. Similarly, hospitalizations and emergency department visits occurred in 0.8% for both groups. Post-procedure pain medications were prescribed in 9.6% of patients undergoing endoscopy pre-liver biopsy versus 13.6% of patients undergoing liver biopsy only.

**Table 3:** Complication rates and additional pain medications administered when compared by BMI (kg/m2), level of training (fellow PGY4 through six versus staff alone) and subjects who underwent endoscopy prior to liver biopsy BMI: Body mass index; PGY: Post-graduate year; ED: Emergency department; Rx: Prescription pain medications; bx: Biopsy. *Chi-square test was performed for comparison of categories. Significance was reported for P value <0.05. **Fisher exact test with significance was reported for cell count <5 for comparison of 2 x 2 categories.

Variables	All complications		Hospitalizations/ED visits		Post-procedure Rx	
	No	Yes	No	Yes	No	Yes
BMI						
≤29	131 (92.3%)	11 (7.7%)	142 (100%)	0 (0%)	120 (84.5%)	22 (15.5%)
30-34	144 (92.9%)	11 (7.1%)	153 (98.7%)	2 (1.3%)	135 (87.1%)	20 (12.9%)
≥35	193 (95.1%)	10 (4.9%)	201 (99.0%)	2 (1.0%)	182 (89.7%)	21 (10.3%)
P value	0.52*		0.42*		0.36*	
Fellows						
No	119 (88.8%)	15 (11.2%)	133 (99.3%)	1 (0.7%)	114 (85.1%)	20 (14.9%)
Yes	349 (95.4%)	17 (4.6%)	363 (99.2%)	3 (0.8%)	323 (88.3%)	43 (11.7%)
P value	0.008*		1.00**		0.34*	
Endoscopy pre-liver bx						
No	349 (93.1%)	26 (6.9%)	372 (99.2%)	3 (0.8%)	324 (86.4%)	51 (13.6%)
Yes	119 (95.2%)	6 (4.8%)	124 (99.2%)	1 (0.8%)	113 (90.4%)	12 (9.6%)
P value	0.39*		1.00**		0.24*	

## Discussion

There were 29 complications (out of 32) that occurred within the one-hour recovery period, providing strong evidence that the majority of complications can be safely identified in the one-hour recovery window. The only hospitalization was also recognized during the first hour of recovery. The subject reported dyspnea and hematemesis and was found to have a pneumothorax that developed minutes after the procedure. Two subjects experienced non-bloody emesis and were managed conservatively with anti-emetics. Three subjects were evaluated in the ED >12 hours after liver biopsy with reports of pain. Small, well-defined subscapular hematomas were seen on CT scan; all subjects were released home from the ED.

We compared our results to the 2004 British Society of Gastroenterology (BSG) guidelines [9]. Our mortality rate was 0% compared to overall mortality of liver biopsy as high as 19% listed in their guidelines. Significant pain occurred in 5% of our patients compared to moderate pain of 3% and severe pain in 1.5% of patients listed in the BSG guidelines, with an overall rate of 30% of patients having post-procedural pain. Fatal hemorrhage was quoted to be 0.11% in the BSG guidelines compared to 0% in our study. Hypotension and vagal episodes occur in 3% of patients according to the BSG guidelines; no episodes were identified in our project. Severe bleeding (Hemoglobin drop of >2 units), hemobilia, and subclinical bleeding (subcapsular and intrahepatic bleeding) were seen as high as 0.5%, 0.05%, and 23%, respectively. In our project, there was no severe bleeding episodes or hemobilia, and our subclinical bleeding rate was 0.6%. Perforation of viscera did not occur in our study and is reported at an incidence of 0.01% to 0.1% in the BSG guidelines.

There is a concern for a higher risk of liver biopsy complications in obese patients. In theory, a larger body habitus creating poor ultrasound windows and a need for deeper needle insertion should make the procedure more complex and challenging; however, we demonstrated that performing a liver biopsy in patients with high BMI is safe with a similar or better rate of complications when compared to non-obese patients. Future prospective studies are needed to confirm our observation.

Some providers might hesitate to perform a liver biopsy after endoscopic interventions due to the risks of bowel perforation in the setting of intestinal distention. Our approach of one-hour recovery post-endoscopic procedure demonstrated to be an effective way to avoid this complication. All patients who underwent an endoscopic procedure were able to obtain their liver biopsy after adequate recovery. Our project demonstrated no difference in terms of complications between patients with and without endoscopic procedure before the liver biopsy. It is possible that the higher doses of sedation used for endoscopic procedures played a role in patients reporting moderate to significant pain post-intervention; however, most patients were widely awake after completing their one-hour post-endoscopy recovery with some patients receiving lower dosing of fentanyl and midazolam for their liver biopsies.

Interestingly, our study showed that not having a fellow involved in the procedure resulted in significantly more complications. We suspect this is related to several aspects of the procedure. Frequently the staff performs the more technically difficult cases such as high BMI, poor ultrasound windows, challenging body habitus, and patients with multiple co-morbidities. It is possible that having two sets of eyes allows for better assessment of the procedure area resulting in a safer intervention.

Frequently we run into the situation where we are not able to reach the liver capsule with our anesthetic needle (1.5 inches) due to excess subcutaneous tissue. To overcome this limitation, we use a spinal needle (3.5 inches) to reach the liver capsule and achieve adequate analgesia. Our study demonstrated that this technique was effective and did not carry a higher risk of complications. It is possible that the deeper application of anesthetic with a spinal needle allowed for better analgesia of the biopsy tract, and as a consequence, decreased the pain level of our patients.

One important finding of our study is that all subjects with a pain level >5 out of 10 were successfully managed in our gastroenterology suite with additional intravenous and/or oral medications. Only 2/61 (3%) of the patients who received Lidocaine alone for the biopsy required post-procedure medications. A small number of patients required an emergency department visit for management of pain more than 12 hours post-liver biopsy, and all of them had an expected finding of a subcapsular hematoma. All these patients were successfully managed in the emergency department and sent home with no subsequent visits.

## Conclusions

Our study demonstrates that percutaneous liver biopsies, using a 14-gauge needle, were overall found to be safe. A one-hour recovery time appears to identify all significant complications and shorten post procedure stay. Our complication rate was comparable and, at times, better than the cited complications in the 2004 BSG guidelines on liver biopsies, notably with recovery times recommended of 3-4 hours in this guideline. The most common complication was pain level above 5, requiring additional medications that prolonged recovery. Lidocaine as an exclusive anesthetic for a liver biopsy is safe and well-tolerated. Morbid obesity or having a pre-procedure endoscopy before a liver biopsy did not increase the risk of complications. A one-hour recovery time could be considered an acceptable recovery time for patients undergoing percutaneous liver biopsies in a gastroenterology suite. Future studies are needed to assess the economic impact and improvement of access to care with a shorter recovery time.
